# A Study of Patients Undergoing Abdominal Hysterectomy in Tertiary Care Institute

**DOI:** 10.7759/cureus.33818

**Published:** 2023-01-16

**Authors:** Anupama V Dhobale, Mangesh G Kohale, Nandkishor J Bankar

**Affiliations:** 1 Obstetrics and Gynaecology, Datta Meghe Medical College, Datta Meghe Institute of Higher Education & Research, Nagpur, IND; 2 Pathology, Datta Meghe Medical College, Datta Meghe Institute of Higher Education & Research, Nagpur, IND; 3 Pathology, Datta Meghe Institute of Higher Education & Research, Nagpur, IND; 4 Microbiology, Jawaharlal Nehru Medical College, Datta Meghe Institute of Higher Education & Research, Wardha, IND

**Keywords:** adenomyosis, dysfunctional uterine bleeding, menstrual abnormality, uterine fibroids, abdominal hysterectomy

## Abstract

Background

Hysterectomy is one of the most common surgical procedures performed.Patient education, compliance, and socioeconomic status are important determinants in choosing the mode of treatment; however, medical treatment is usually inadequate.Thus, in the present study, we tried to study the various profiles of patients undergoing abdominal hysterectomies reported in tertiary care centers.

Materials and Methods

Brief clinical data were noted from the case records, which include age, parity, presenting symptoms, past surgical and medical history, hemoglobin status, preoperative dilatation and curettage, and indications of hysterectomy.

Results

Most hysterectomies were performed on women between the ages of 41 and 45, with the average parity being 3.2 and the majority of cases having three children. Abnormal vaginal bleeding associated with various forms of menstrual irregularities was the most common complaint for which patients sought advice, and the incidence of patients undergoing tubal ligation was the most common previous surgery procedure, with anemia being the most common preoperative-associated condition. The fibroid was the most common demonstration in the current study, which brought forth abdominal hysterectomy for the chosen participants.

Conclusion

Findings from the current study suggest abdominal hysterectomy to be the most preferred route of surgery commonly in the women of age group 40 to 45 years or higher, precisely due to the occurrence of a higher parity rate in this age category. Fibroid uterus, dysfunctional uterine bleeding, and the presence of other menstrual complaints were the most probable indications for abdominal hysterectomy demonstrated by the individuals in the present study. Moreover, anemia was the most predominant complication associated pre-operatively, followed by other clinical manifestations such as hypertension, diabetes mellitus, and urinary tract infection.

## Introduction

Hysterectomy is one of the most frequent surgical treatments. It is the second most frequently performed major surgical surgery in developed nations after cesarean delivery [1]. Menstrual abnormalities are a frequent reason for women to present at gynecological outpatient clinics. Women require timely, safe, and effective treatment of menstrual problems. Conservative management includes medical treatment and endometrial ablation. 

Patient education, compliance, and socioeconomic status are important determinants in choosing the mode of treatment choices. Medical treatment is usually inadequate and often disappears. Hysterectomy is an effective treatment for these patients; on the other hand, it provides such women with immediate and radical relief from their distressing problems. Uterine leiomyomas consistently rank as the primary reason for hysterectomy in practically all studies, which implies that the indications shift with the patient’s age, as anticipated [2]. The majority of patients who underwent surgery were women between the ages of 30 and 55 [3]. 

## Materials and methods

Between December 2010 and October 2012, a rural teaching hospital’s Department of Obstetrics and Gynecology undertook this prospective study on hysterectomy and its associated consequences. The following inclusion and exclusion criteria were adopted for this study. Patients who demonstrated requisite indications and were scheduled for abdominal hysterectomy were included in this study: patients who underwent abdominal hysterectomy after a failed vaginal hysterectomy and those who underwent a vaginal and obstetrical hysterectomy.

There were a total of 438 patients in the study duration who had undergone hysterectomy. According to the aforementioned inclusion and exclusion criteria, 288 cases were disqualified because 287 of them had undergone vaginal hysterectomy, and one case of fibroid had been initially scheduled for a vaginal hysterectomy but had to undergo abdominal hysterectomy due to the patient's large fibroid uterus, which prevented vaginal delivery. Therefore, this study included 150 patients who underwent abdominal hysterectomies. From the case records of patients who underwent an abdominal hysterectomy, a few clinical details were noted, and their relationships with age, parity, general physical health before surgery, indications, and complications were studied. Patients received attentive preoperative monitoring and quality treatment throughout their hospital stay. Individuals were monitored during their hospital stay, as this study focused on the characteristics of patients who underwent hysterectomies.

Additionally, an attempt was made to gauge patients’ readiness for participation in the study. Following the initial appointment, a complete medical history was obtained. Before surgery, important medical history and information on gynecological care were documented. Baseline examinations were conducted, including chest radiography, ECG, hemoglobin estimation, blood grouping with RH type, standard urine examination, blood sugar, liver function tests, kidney function tests, blood pressure, and abdominopelvic ultrasound. The presence of any other ailments, such as diabetes mellitus, hypertension, and ischemic heart disease, was managed in conjunction with the medical staff. In cases of severe anemia, preoperative anemia was treated with blood transfusions or injectable iron. In all cases of postmenopausal bleeding and a few suspected patients with abnormal bleeding, preoperative curettage was performed to rule out malignancies, and the cases were studied only after the diagnosis was made.

Before deciding on the type of anesthesia, each patient was thoroughly analyzed by the anesthetist. The type of anesthesia administered was decided only by the anesthetist. The type of incision was chosen by the operator. The decision to remove the ovaries was made by the surgeon depending on the age of the patient and the appearance of the ovaries. Blood loss was determined by counting the number of operations used during the procedure, which was one of the intraoperative complications. The mops used in this investigation measured 34 cm × 24 cm. The ballpark figure for blood loss on an average estimated 1/4th of soaked mops contained 20 ml, ½ soaked 40 ml and fully soaked 100 ml of blood. Postoperatively, all patients were meticulously followed up. Postoperative complications, such as fever, urinary tract infections, vaginal cuff cellulitis, and respiratory and abdominal wound infections, were noted. 

An antibiotic coverage injection of cefotaxime was administered to all patients, intravenous (IV) prophylactic single preoperative dose, half an hour prior to surgery, and postoperative IV for the first three days and thereafter oral cephalosporins for a further 4 to 5 days until the patient was discharged. Operative patient documents were preserved, and patients were discharged between the seventh and ninth postoperative days after suture removal after receiving the histopathology report. All patients were treated by early ambulation and were encouraged within their limits of physical capacity to become independent as soon as possible.

Statistical analysis was performed using Microsoft Excel (Redmond, USA) to determine the mean and percentage of the parameters that include age-wise distribution, parity, past surgical and medical history, hemoglobin percentage on admission, frequency of pre-operative dilatation and curettage, and indications of hysterectomy.

## Results

It was found that women between the ages of 41 and 45 made up the majority of patients having hysterectomies. The utero-vesical fistula-affected 25-year-old woman with Youssef's syndrome was the youngest patient observed, whereas the oldest was affected with fibroid. The mean age for abdominal hysterectomy was 41.64±7.21yrs. The mean parity for the abdominal hysterectomy was 3.26 ± 0.97 (Table 1).

**Table 1:** Age-wise distribution in study patients

		No. of abdominal hysterectomy	Percentage
Age	21-25	01	0.67%
	26-30	03	02%
	31-35	30	20%
	36-40	34	22.67%
	41-45	59	39.33%
	46-50	11	7.33%
	More than 50	12	08%
Parity	P1	03	02%
	P2	30	20%
	P3	62	41.34%
	P4	41	27.33%
	P5	11	7.33%
	P6	03	02%
	Total	150	100%

Menstrual issues accounted for 68.65% of the patients' complaints, followed by additional issues such as abdominal discomfort (63.33%) and leucorrhoea (37.33%), which were often reported. Before performing a hysterectomy, 7.33% of patients who reported postmenopausal bleeding underwent a comprehensive investigation and had cancer ruled out (Table 2).

**Table 2:** Symptom-wise distribution in study group

Symptom		No. of patients*	Percentage
Menstrual disturbances	Menorrhagia	56	37.33%
	Metrorrhagia	20	13.33%
	Polymenorrhoea	07	04.66%
	Dysmenorrhoea	19	12.66%
	Amenorrhoea	01	00.67%
Pain in abdomen		95	63.33%
Leucorrhoea		56	37.33%
Post-menopausal bleeding		11	07.33%
Backache		09	06.00%
Urinary complaints		07	04.67%
Lump in abdomen		04	02.67%
Pruritus vulvae		05	03.33%
Dyspareunia		01	00.67%
Bowel complaints		06	04.00%

It was seen that tubectomy was the most commonly performed previous surgery (85.33%); out of that, 01.33% had laparoscopic tubal surgery, and 84% had an abdominal tubal ligation. 2.67% of patients had undergone appendicectomy, while 6.67 % of patients had a cesarean section, and no surgical history was seen in 10% of patients. It was observed that anemia was the most predominant pre-operative associated problem in the present study (17.33%), followed by hypertension which constituted 4.6%, diabetes mellitus (3.33%), urinary tract infection, and IHD in 4% of the hysterectomy population (Table 3).

**Table 3:** Distribution of patients with past surgical and medical history

Type of surgery	No. of patients	%
Tubectomy	128	85.33%
Appendicectomy	04	02.67%
Lower segment caesarean section (LSCS)	10	06.67%
Laparotomy	04	02.67%
No surgery	15	10.00%
Disease	No. of patients (n= 150)	%
Anemia	26	17.33%
Hypertension	07	4.66%
Diabetes Mellitus (DM)	05	3.33%
Urinary tract infection	06	4%
Ischemic Heart Disease (IHD)	06	4%
Pulmonary Tuberculosis	03	02%
Asthma	01	0.67%
Incisional hernia	01	0.67%
Hyperthyroidism	01	0.67%

From (Table 4) it was observed that there were eight patients (5.33%) with Hemoglobin less than 8 gm%. 82.67% had hemoglobin more than 10 gm%. Hemoglobin levels where required were increased either by blood transfusion or by IV iron infusion.

**Table 4:** Distribution of patients with Hemoglobin (gm%) on admission

Hemoglobin (gm %)	No. of patients	%
<8	08	05.33%
8 to 10	18	12.00%
>10	124	82.67%
Total	150	100%

Table 5 shows that preoperative Dilatation & Curettage was done in 13.33% of cases. Fractional curettage was done in all the patients with post-menopausal bleeding. 

**Table 5:** Frequency of pre- operative Dilatation & Curettage D & C: Dilatation and Curettage

D & C	No.	Percentage
Done	20	13.33
Not done	130	86.67

In the current study, it emerged that Fibroid Uterus, which accounted for roughly 30% of hysterectomy indications, was followed by Dysfunctional Uterine Bleeding (28%), Adenomyosis (11.34%), and Chronic Cervicitis (9.33%) (Table 5).

**Table 6:** Distribution of patients according to the indications of hysterectomy DUB: Dysfunctional uterine bleeding, PID: Pelvic inflammatory disease

Sr. no.	Indication	No.	Percentage
1.	Fibroid	45	30%
2.	DUB	42	28%
3.	Adenomyosis	17	11.34%
4.	Chronic cervicitis	14	9.33%
5.	Polyp	09	06%
6.	Ovarian cyst/tumor	06	04%
7.	Chronic PID	05	3.33%
8.	Post-menopausal bleeding	05	3.33%
9.	Adnexal mass/Tubo Ovarian (TO) mass	02	1.33%
10.	Endometriosis	01	0.67%
11.	Malignancy	02	1.33%
12.	Dermoid cyst	01	0.67%
13	Menouria	01	0.67%
	Total	150	100%

## Discussion

The present study was conducted in the Department of Obstetrics and Gynecology of a hospital in India, with the objective of studying the profile of patients undergoing abdominal hysterectomy. In contrast to the studies conducted by Amirikia et al. [3] (37%) and Clarke et al. [4] (30%), the greatest number of hysterectomies were performed in the age range of 41 to 45 years (39.33 %). The mean age of hysterectomy in the present study was 41.64±7.21yrs, which was comparable with Susan M Taylor et al. [5] (42.20yrs), Tariq Miskry et al. [6] (42yrs) and Sunanda Bharatnur et al. years [7] (44.4yrs). 

These findings suggest that women agree to hysterectomy at this stage of their lives because they have stopped having children and feel that it will resolve their issues. When a patient is under 30 years old, and particularly when they are under 25, surgeons are very hesitant and apprehensive about suggesting a hysterectomy. However, one instance of a 25-year-old woman who had a uterovaginal fistula along with Youssef's syndrome and menstrual irregularities, as well as a history of two LSCS and one hysterectomy, was observed during this study. All three patients were 30 years of age and had undergone hysterectomies after completing their families. One patient developed gonococcal salpingitis, which led to recurrent episodes of PID. Among the other two patients, one had chronic cervicitis that was unresponsive to antibiotics, and the other had DUB that did not respond to conservative therapy. Due to our preference for a liberal outlook at this age, 15.33% of the incidents fall into the age category of those over 45. These findings are comparable to those reported by J. Ravindran et al. [8] (3.7), Bharatnur et al. [7] (3.6), and Taylor et al. [5] (3.6) in that the majority of cases (41.34%) were having three children with a mean parity of 3.2. 

Most patients present with more than one symptom. Abnormal vaginal bleeding associated with various forms of menstrual irregularities was the most common complaint for which patients sought advice (68.65%), comparable to that reported by Dicker et al. 68.65%, which is comparable to that reported by Dicker et al. [9] (58%).

The total incidence of menstrual irregularities, menorrhagia, metrorrhagia, polymenorrhagia, and dysmenorrhea was 68.65%, 37.33%, 13.33%, 4.66%, and 12.66%, respectively. Postmenopausal bleeding was observed in 07.33% of the patients. Menorrhagia (69%), dysmenorrhea (24%), and postmenopausal hemorrhage (5.5%) were the symptoms mentioned in the investigation of all the patients who underwent abdominal hysterectomy. Menorrhagia was the most common symptom among the patients who underwent a hysterectomy. This could be attributed to the fact that most of the patients visiting this institute were of lower economic strata and usually did not opt for conservative procedures but were insistent to undergo hysterectomies instead. 

Abdominal or pelvic pain was the next most common complaint observed, comprising 63.33%. Pain is usually associated with dysmenorrhea, abdominal lumps, or generalized intractable pain. Compared with the current study (63.33%), the incidence of pain reported in other studies was lower. In the present study, a hysterectomy was not performed for pain alone. This is always associated with other complaints. Patients with pelvic inflammatory disease, which is an important cause of pain in the lower abdomen, were included in this study.

The next most common complaint was leucorrhea in 37.33% of patients. The most common causes of leukorrhea are cervical erosion and cervicitis. It was observed that 4.67% presented with urinary symptoms in the form of burning micturition, frequency of micturition, or stress incontinence. The history of surgical procedures was also evaluated. In the present study, a history of appendectomy was present in four patients (2.67%), lower segment cesarean section (LSCS) in 10 patients (6.67%), and previous laparotomy in four patients (2.67%). Fifteen patients (10%) had no surgical history. While studying the associated medical conditions, it was observed that anemia was the most common preoperative-associated condition (17.33%), followed by hypertension (4.66%), ischemic heart disease (4%), urinary tract infection (4%), and diabetes mellitus (3.33%). 

The most probable reason for abdominal hysterectomy in the current study was fibroids (30%), which is comparable to the findings of Bharatnur et al. [7] (31%) and Dicker et al. [9] (40.2%) and Farquhar et al. [10] (40%). According to Amirikia et al. [3] and YGS Chang et al. [11], the incidence of fibroids in abdominal hysterectomy is 76% and 55%, respectively. In the current analysis, dysfunctional uterine bleeding was the second most prevalent reason for the hysterectomy, accounting for 28% of cases, which was similar to the rate reported by Bharatnur et al. [7] (20%). Dicker et al. [9] reported a lower incidence of DUB (17%) than that reported by Jennifer et al. [12] (14.9%) and Amirikia et al. [3] (7.7%).

Adenomyosis represented 11.34% of the hysterectomy indications in the current study. Nisha et al. reported an adenomyosis incidence of 6.5%. [13] and 5.7% by YGS Chang et al. [11], 5.5% by Ravindran et al. [8], and 4% by Bharatnur et al. [7] among abdominal hysterectomy patients. Fourteen patients had persistent cervicitis, representing 9.33% of those who required a hysterectomy. 

Antibiotics were administered to individuals with persistent leucorrhea over an extended period. However, as menopause approached, more hysterectomies were performed because of the increased concern that cancer would develop in the infected cervix. Without a hysterectomy, each patient would require extensive follow-up care over an extended period, which would have been expensive. Additionally, because these patients are poor and come from far-flung villages in a nation such as India, they may not have returned for regular checkups. This likely explains why hysterectomy is frequently performed for benign persistent cervicitis.

The rate of persistent cervicitis in the current study is 9.33%. Nisha et al. [13] reported that the incidence of chronic cervicitis is 2.5%. This was ascribed to low socioeconomic status, recurrent childbearing, and early marriage. The incidence of chronic cervicitis was reported to be 32% by Sunanda Bharatnur et al. [7], which is higher than the current study's 9.33 %. In the current study, 4% of hysterectomies were performed for ovarian tumors, of which one patient had a dysgerminoma, while five individuals had benign ovarian tumors removed by surgery. One patient with a dermoid cyst complained of menopausal bleeding when she first visited the doctor.

In the present study, five patients (3.33%) were operated on for chronic PID, which was comparable with the incidence reported by YGS Chan et al. [11] as 2.8%. Ravindran et al. [8] reported the incidence of chronic PID in 1.5% of patients and Kapoor Nisha et al. [13] in 2.5% of patients. In such cases, abdominal hysterectomy is recommended because it is challenging to predict the severity of adhesions in anticipation. In the present study, nine patients (6%) had cervical or endometrial polyps and underwent abdominal hysterectomies. Five patients (3.33%) were postmenopausal and underwent an abdominal hysterectomy after confirming that there was no malignancy. 

In the present study, two patients (1.33%) underwent surgery for a tubo-ovarian mass. A small group of tubo-ovarian infections that do not respond to conservative treatment, where large adnexal masses persist and continue to give rise to symptoms, require surgical removal. The incidence quoted by YGS Chan et al. 18 as 5.7%, Kapoor Nisha et al. [13] as 10.5%, and J Ravindran et al. [8] as 5.7% for adnexal mass. Two patients (1.33%) had malignancies and presented with post-menopausal bleeding. One cervical cancer stage IIA was found on D/C with fractional curettage, while the other endometrial carcinoma stage IB underwent Wertheim's hysterectomy. According to Chan et al. [11] and Ravindran et al. [12], the incidence of malignancy was 4%, 6.9%, and 9%, respectively, according to YGS Chan et al. [11] and J Ravindran et al. [8].

In the present study, one patient (0.67%) underwent surgery for endometriosis. She underwent an abdominal hysterectomy with a presacral neurectomy. Fortunately, endometriosis is not a common problem in our country, as customs of early marriage and childbearing still prevail in most communities. Incidences reported by other authors for endometriosis were by YGS Chan et al. [11] at 7.9%, by Clarke et al. [4] at 7.4%, and by Nisha et al. [13] at 16.5%, respectively. 

## Conclusions

One hundred fifty instances of abdominal hysterectomies were examined for the current study in order to determine the probable indications demonstrated by the individuals who underwent abdominal hysterectomies and their rationale. It was observed from the present study that 54.66% of patients were above the age of 40 years, with the most common age group for hysterectomy being 41-45 years, for which fibroids were the most common indication, followed by dysfunctional uterine bleeding. Higher parity was also observed to favor hysterectomy. Most patients presented with menstrual complaints along with pelvic pain. In patients who underwent previous surgeries and laparotomy, this was the best route preferred.
